# The Impact of the SARS-CoV-2 Pandemic on Stress and Anxiety of Dental Students

**DOI:** 10.1055/s-0042-1760299

**Published:** 2023-06-05

**Authors:** Andreas Zenthöfer, Andreas Graf, Peter Rammelsberg, Anna-Luisa Klotz

**Affiliations:** 1 Department of Prosthodontics, Dental School, University of Heidelberg, Heidelberg, Germany

**Keywords:** stress, dentistry students, COVID-19

## Abstract

**Objectives** To investigate the impact of the severe acute respiratory syndrome-coronavirus-2 (SARS-CoV-2) pandemic on stress and anxiety of preclinical and clinical dental students.

**Materials and Methods** Dental students (participants) in their clinical course (CC; *n* = 64) or preclinical course (PCC; *n* = 53) were included in the study. The subjective perceived levels of stress and anxiety were evaluated using the Dental Environment Stress (DES) questionnaire and the Depression, Anxiety and Stress Scales (DASS) questionnaire. Cortisol levels were measured in saliva samples collected from participants. Knowledge of the pandemic was evaluated using a 100-mm visual analog scale. All data were collected twice: once during the university holidays and once during term time.

**Statistical Analysis** Results from DES, DASS, and salivary cortisol tests were compared between baseline and follow-up using descriptive and bivariate statistics. Multivariate linear regression models were computed with DES, DASS, and cortisol values as dependent variables to analyze possible influencing factors.

**Results** Participants showed medium levels of stress and anxiety at baseline and follow-up. The DASS score in the “anxiety” subdomain was significantly higher in the PCC group than in the CC group at baseline ( *p* < 0.001) and increased during term time. DASS scores in the “stress” subdomain also increased during term time. However, both subdomain scores were lower than the cutoff value for a psychological disorder. The mean total DES scores were 615.9 ± 97.7 in the CC group and 580.40 ± 98.9 in the PCC group. These scores indicated medium stress levels and were not significantly different between the groups, nor did they change during the study period. Mean saliva cortisol levels were higher in the CC group (9.2 ± 5.2) than in the PCC group (4.9 ± 2.2) at baseline ( *p* < 0.001) but converged by follow-up. Multivariate regression models showed that intraindividual perception of stress at baseline was consistently the most important aspect for changes in stress and anxiety levels during term time. The SARS-CoV-2 pandemic affected stress and anxiety levels in some participants, but this was not ubiquitous.

**Conclusion** Intraindividual differences in stress perception seem to be more relevant than course affiliation (preclinical or clinical) or the SARS-CoV-2 pandemic to stress and anxiety levels in dental students.

## Introduction

Dentistry is a stressful profession, with 86% of dentists reporting sustained medium to high levels of stress. 1 Stress increased physical or emotional tension and strain. It can negatively affect health, 2 and lead to psychological problems, such as depressive disorders, anxiety, obsessive–compulsive disorder, and burnout. 3 Studies have shown that dental students experience significant stress symptoms while studying. 4 5 6 7 8 9 10 This stress is caused by several factors, including pressure to perform, time pressure, first contact with patients, problems with teaching staff, and financial difficulties. 11 Consequently, dental students suffer more from anxiety, obsessive-compulsive disorders, and depression than adults of the same age do. 12 13 14 15 This problem is predominant during the clinical studies. 8 16 In this group, 10% of students suffer from emotional exhaustion, 17% from severe lack of performance, and 28% from depersonalization. 16 These psychological problems also reduce efficiency during working and learning. 17

The severe acute respiratory syndrome coronavirus-2 (SARS-CoV-2) pandemic may have been an additional source of stress for dental students during their studies. SARS-CoV-2 causes coronavirus disease 2019 (COVID-19), 18 and infection usually takes place via droplet transmission, putting employees in health care and dentistry at a higher risk of infection. 19 Various measures have been implemented to prevent the virus from spreading among health care professionals, 19 and these measures have affected dental students. Safety measures included periodic testing and provision of suitable protective equipment. These changes present challenges, adjustments, and uncertainties, which may cause additional stress and anxiety among dental students. However, this has not been investigated thematically.

The aim of this study was to examine the impact of the SARS-CoV-2 pandemic on stress and anxiety in dental students at the University of Heidelberg by measuring changes in objective and subjective indicators of stress during the holidays (baseline) and during term time (follow-up). We hypothesized that stress levels at baseline would be associated with increased stress levels during term time.

## Materials and Methods

### Study Setting

This longitudinal cohort study was approved by the local review board of the University of Heidelberg (approval number S-627/2020). The study was registered in the German Register for Clinical Studies (DRKS00023499) and was performed at the Dental School of the University of Heidelberg, Baden-Württemberg, Germany. All dental students in their second and fourth years of study were invited to participate in the study and give written consent. There were no other inclusion criteria. In total, 117 students agreed to participate—64 students from the clinical course (CC group) and 53 students from the preclinical course (PCC group).

Sociodemographic data were recorded and participants were asked to complete validated questionnaires to determine stress and anxiety levels and knowledge of the SARS-CoV-2 pandemic. Salivary cortisol levels were also measured. The questionnaires and salivary cortisol tests were conducted once during the holidays before the course started (baseline) and again during term time (follow-up). To protect participants, suitable measures were taken, such as maintaining social distancing and wearing protective equipment during cortisol tests. SARS-CoV-2 tests were also performed to make sure that participants were not infected.

### Level of Knowledge and Stress Triggered by the SARS-CoV-2 Pandemic

To subjectively evaluate knowledge of the pandemic and stress triggered by the pandemic, we asked three questions, each of which was answered on a scale of 0 to 100 (0 = never/low, 100 = very often, very high). The questions were: (1) How often do you inform yourself about the current status of the pandemic? (2) How would you rank your level of knowledge of SARS-CoV-2, which causes COVID-19? and (3) Do you feel more stress in your studies because of the pandemic? There was one additional question (Do you feel stress when you think about the course?), which received a yes/no answer. We also administered a questionnaire that assessed the level of knowledge of the pandemic. The questionnaire contained 11 questions and was adapted from the guidelines for medical staff published by Modi et al and translated into German. 20

### Stress and Anxiety Measurements

Stress was evaluated in participants using the German version of the Dental Environment Stress (DES), which has been described as a reliable and valid tool for measuring stress. 21 22 The DES contains 25 items representing various stressors, which are divided into seven subdomains: faculty and administration (questions 9, 12, 18), academics (questions 1–4), manual skills (questions 6, 10), financial obligations (question 21), patient care (questions 5, 7, 8, 11), personal problems (questions 13–17, 22, 25), and family (questions 19, 20, 22–24). Students were asked to score each item on a 5-point Likert scale (not stressful = 10, highly stressful = 50), so the total DES score ranged from 250 to 1,250 points. Sum scores were also calculated for the seven subdomains ( Table 1 ).

**Table 1:** Original version of the Dental Environment Stress questionnaire 21 22

	Stress factors in the dental educational environment	1	2	3	4	5
1	Stress due to amount of classwork					
2	Stress due to difficulty of classwork					
3	Stress due to examinations and grades					
4	Stress due to peer competition					
5	Stress due to patient care responsibilities					
6	Stress due to difficulty in learning clinical procedures					
7	Stress due to patients' attitudes toward me					
8	Stress due to patients' attitudes toward dentistry					
9	Stress due to atmosphere created by clinical professors					
10	Stress due to difficulty in learning precision manual skills required in preclinical and laboratory practices					
11	Stress due to reliability of professional dental laboratories in prompt return of cases					
12	Stress due to administrative responses to student needs					
13	Stress due to roommate relationships					
14	Stress due to dating relationships					
15	Stress due to alcohol usage					
16	Stress due to drug usage					
17	Stress due to reconsideration of dentistry as proper career choice					
18	Stress due to fear of flunking out of school					
19	Stress due to marriage relationship					
20	Stress due to child care					
21	Stress due to financial responsibilities					
22	Stress due to personal physical health					
23	Stress due to physical health of other family members					
24	Stress due to parent–student relationship					
25	Stress due to other personal problems					

Note: Stress factors in the dental educational environment. Please rate level of stress factors in range from least stressful (1) to very stressful (5).

Anxiety was assessed in participants using the short version of the Depression, Anxiety and Stress Scales (DASS). 23 The DASS contains 21 items related to depression (seven questions), anxiety (seven questions), and stress (seven questions). Participants scored each item on a 4-point Likert scale (0 = did not apply to me at all, 1 = applied to me to some degree or some of the time, 2 = applied to me to a considerable degree or a good part of time, and 3 = applied to me very much or most of the time). The total score ranged from 0 to 21. Scores of ≥ 10 were indicative of depression and stress, and scores of ≥ 6 were indicative of anxiety ( Table 2 ).

**Table 2:** Depression, Anxiety and Stress Scales questionnaire 23 32

1	I found it hard to wind down	0	1	2	3
2	I was aware of dryness of my mouth	0	1	2	3
3	I could not seem to experience any positive feeling at all	0	1	2	3
4	I experienced breathing difficulty (e.g., excessively rapid breathing, breathlessness in the absence of physical exertion)	0	1	2	3
5	I found it difficult to work up the initiative to do things	0	1	2	3
6	I tended to overreact to situations	0	1	2	3
7	I experienced trembling (e.g., in the hands)	0	1	2	3
8	I felt that i was using a lot of nervous energy	0	1	2	3
9	I was worried about situations in which I might panic and make a fool of myself	0	1	2	3
10	I felt that I had nothing to look forward to	0	1	2	3
11	I found myself getting agitated	0	1	2	3
12	I found it difficult to relax	0	1	2	3
13	I felt downhearted and blue	0	1	2	3
14	I was intolerant of anything that kept me from getting on with what I was doing	0	1	2	3
15	I felt I was close to panic	0	1	2	3
16	I was unable to become enthusiastic about anything	0	1	2	3
17	I felt I was not worth much as a person	0	1	2	3
18	I felt that I was rather touchy	0	1	2	3
19	I was aware of the action of my heart in the absence of physical exertion (e.g., sense of heart rate increase, heart missing a beat)	0	1	2	3
20	I felt scared without any good reason	0	1	2	3
21	I felt that life was meaningless	0	1	2	3

Note: Please read each statement and circle a number 0, 1, 2, or 3 which indicates how much the statement applied to you over the past week. There are no right or wrong answers. Do not spend too much time on any statement. The rating scale is as follows: 0—Did not apply to me at all. 1—Applied to me to some degree, or some of the time. 2—Applied to me to a considerable degree, or a good part of time. 3—Applied to me very much, or most of the time.

### Saliva Cortisol Levels

To evaluate stress objectively, we measured cortisol levels in participants' saliva. As cortisol levels fluctuate during the day, saliva samples were collected between 12:00 a.m. and 1:00 p.m. Saliva samples were collected using Cortisol Salivettes (SARSTEDT AG & CO.; Nümbrecht, Germany). Participants were asked to rinse their mouths with water for 10 minutes before taking the saliva sample. After rinsing, participants chewed a cotton roll for 45 to 60 seconds and placed it in the Salivette. The Salivettes were then hermetically sealed and sent on the same day to the test laboratory (Daacro GmbH & Co. KG; Trier, Germany) for evaluation. Cortisol levels were measured in nanomole/liter.

### Statistical Evaluation

Mean values ± standard deviations (SD), counts ( *n* ), and frequencies (%) were used to present baseline and follow-up data. Results were given as means ± SD or counts (%). Pairwise comparisons were performed using *t* -tests and chi-squared tests. Multivariate regression analyses were performed to detect possible influences of confounders on dependent variables (DES, DASS-S, and DASS-A scores, and cortisol values). Bivariate associations were considered significant at *p* < 0.05.

All statistics were calculated using SPSS version 22.0 (IBM Corporation; New York, United States). The *p* -values of less than 0.05 were regarded as statistically significant.

## Results

### Participants Characteristics

Out of the 165 dental students who were invited to participate in the study, 117 ( *n* = 64 in the CC group and *n* = 53 in the PCC group) gave their informed consent (response rate 70.9%). The mean ± SD age was 24.7 ± 2.7 years in the CC group and 22.9 ± 3.2 years in the PCC group. Thirty participants in the CC group (46.9%) and 21 participants in the PCC group (37.7%) were male. Participants in the CC group informed themselves significantly more often about the coronavirus pandemic than participants in the PCC group did ( *p* = 0.001). Furthermore, participants in the CC group felt more stress at baseline because of the coronavirus pandemic than participants in the PCC group did ( *p* < 0.001). The questionnaire on knowledge of the coronavirus revealed no differences between the groups at baseline but significantly more knowledge in the PCC group than in the CC group at follow-up (CC: 73.6 ± 7.9 and PCC: 78.6 ± 10.2; *p* = 0.003).

Scores for the anxiety subdomain in the DASS questionnaire were significantly higher in the PCC group than in the CC group at baseline (CC: 2.6 ± 2.8 and PCC: 4.9 ± 4.2; *p* < 0.001) and increased at follow-up (CC: 4.0 ± 3.3 and PCC: 6.0 ± 4.4), but slightly missed the cutoff value for a psychological disorder. Similarly, scores for the stress subdomain were also higher in the PCC group than in the CC group at baseline (CC: 5.2 ± 4.0 and PCC: 7.4 ± 4.3) and also increased at follow-up (CC: 8.5 ± 4.2 and PCC: 8.4 ± 4.8), but also missed the cutoff value for a psychological disorder.

Several subdomains of the DES questionnaire differed significantly between the study groups, but there was no difference in the mean total DES score between the CC group (615.9 ± 97.7) and the PCC group (580.4 ± 98.9), which indicated medium stress levels in both the groups ( *p* > 0.05). In addition, the total score did not change remarkably during study period ( Tables 3 and 4 ).

**Table 3:** Descriptive statistics for participant characteristics at baseline

	Clinical course, mean (SD) or frequency (%)	Preclinical course, mean (SD) or frequency (%)	*p* -Value
Age	24.7 (2.7)	22.9 (3.2)	**< 0.001**
Gender (male)	30 (46.9%)	21 (37.7%)	0.431
Information about COVID-19	71.3 (21.5)	61.9 (23.1)	**0.001**
Stress due to COVID-19	62.7 (21.8)	41.9 (28.3)	**< 0.001**
Stress when thinking about the course	50 (78.1%)	43 (81.1%)	0.947
Cortisol level, nmol/L	9.2 (5.2)	4.9 (2.2)	**< 0.001**
DASS-A	2.6 (2.8)	4.9 (4.2)	**< 0.001**
DASS-S	5.2 (4.0)	7.4 (4.3)	0.067
Questionnaire about COVID-19	77.6 (11.2)	77.3 (11.0)	0.083
Total score DES	615.9 (97.7)	580.4 (98.9)	0.055
DES subdomains			
Faculty and administration	106.6 (20.3)	92.5 (20.5)	**0.001**
Academics	122.3 (25.3)	131.9 (22.3)	**0.027**
Manual skills	56.1 (14.4)	62.5 (18.2)	**0.002**
Financial obligations	23.9 (11.2)	23.4 (11.9)	0.064
Patient care	115.5 (27.0)	81.9 (36.5)	**< 0.001**
Personal problems	123.4 (34.7)	128.3 (43.7)	0.095
Family	93.9 (33.6)	86.8 (26.7)	0.268

Abbreviations: COVID-19, coronavirus disease 2019; DASS-A, Depression, Anxiety and Stress Scales anxiety; DASS-S, DASS stress; DES, Dental Environment Stress; SD, standard deviation.

Note: Data are presented as means (SD) or counts (frequency) (clinical course, *n* = 64; preclinical course, *n* = 53). Significant *p* values are marked in bold.

**Table 4:** Descriptive statistics for participant characteristics at follow-up

	Clinical course, mean (SD) or frequency (%)	Preclinical course, mean (SD) or frequency (%)	*p* -Value
Age	24. 9 (2.7)	22.91 (3.24)	**< 0.001**
Gender (male)	30 (46.9%)	21 (37.7%)	0.431
Information about COVID-19	62.1 (21.0)	50.4 (24.2)	**0.006**
Stress due to COVID-19	1.3 (0.5)	1.3 (0.4)	0.698
Stress when thinking about the course	45 (70.3%)	39 (73.6%)	0.695
Cortisol level, nmol/L	8.1 (5.1)	7.2 (4.6)	0.327
DASS-A	4.0 (3.3)	6.0 (4.4)	**0.008**
DASS-S	8.5 (4.2)	8.4 (4.8)	0.982
Questionnaire about COVID-19	73.6 (7.9)	78.6 (10.2)	**0.003**
Total score DES	594.2 (141.6)	571.1 (117.5)	0.345
DES subdomains			
Faculty and administration	108.9 (58.3)	92.0 (23.2)	**0.048**
Academics	125.5 (25.8)	130.2 (24.1)	0.313
Manual skills	51.1 (15.4)	58.7 (19.0)	**0.019**
Financial obligations	19.5 (11.1)	25.9 (13.2)	**0.006**
Patient care	101.7 (33.8)	67.9 (35.2)	**< 0.001**
Personal problems	121.4 (39.8)	134.7 (48.1)	0.104
Family	90.5 (32.0)	88.5 (31.4)	0.738

Abbreviations: COVID-19, coronavirus disease 2019; DASS, Depression, Anxiety and Stress Scales; DASS-A, DASS anxiety; DASS-S, DASS stress; DES, Dental Environment Stress; SD, standard deviation.

Note: Data are presented as means (SD) or counts (frequency) (clinical course, *n* = 64; preclinical course *n* = 53). Significant *p* values are marked in bold.

Mean salivary cortisol levels were significantly higher ( *p* < 0.001) in the CC group at baseline (9.2 ± 5.2) than in the PCC group (4.9 ± 2.2), indicating medium stress levels in the CC group. This difference between the groups was no longer significant at follow-up ( *p* > 0.05) ( Tables 3 and 4 ).

### Multivariate Analysis

Multivariate analysis with cortisol levels as the dependent variable confirmed the bivariate analysis. The baseline cortisol level was the most important determinant for changes in cortisol levels between baseline and follow-up (C: −0.859; *p* < 0.001). Participants with higher cortisol levels at baseline had a higher increase in cortisol levels at follow-up, whereas clinical or preclinical studies had no effect on cortisol levels (C: 0.288; *p* = 0.777) ( Table 5 ).

**Table 5:** Multivariate regression analysis with changes of cortisol level as the dependent variable and target variables at baseline

Variable	C	95% CI LB	95% CI UB	*p* -Value
Change in cortisol level				
Course (clinical course)	0.288	−1.727	2.304	0.777
Cortisol level	−0.859	−1.075	−0.642	**< 0.001**

Abbreviations: C, regression coefficient; CI, confidence interval; LB, lower boundary; UB, upper boundary.

Note: Significant *p* -values are marked in bold.

Multivariate analysis with DES scores as the dependent variable showed that knowledge of COVID-19 (C: −0.895; *p* = 0.009) and the baseline DES score (C: 0.575; *p* < 0.001) significantly affected changes in the DES score at follow-up, confirming the bivariate analysis ( Table 6 ). Higher stress levels at baseline further increased the DES score at follow-up and vice versa. Knowledge of the pandemic had the opposite association with DES scores at follow-up.

**Table 6:** Multivariate regression analysis with changes in DES score as the dependent variable and target variables at baseline

Variable	C	95% CI LB	95% CI UB	*p* -Value
Change in DES score				
Information about COVID-19	−0.895	−1.561	−0.229	**0.009**
DES score	0.575	0.460	0.690	**< 0.001**

Abbreviations: C, regression coefficient; CI, confidence interval; COVID-19, coronavirus disease 2019; DES, Dental Environment Stress; LB, lower boundary; UB, upper boundary.

Note: Significant *p* -values are marked in bold.

Factors that increased the DASS stress score at follow-up were stress when thinking during the course (C: 2.555; *p* = 0.003), stress due to COVID-19 (C: 0.036; *p* = 0.004), and the baseline DASS stress score (C: 0.515; *p* < 0.001) ( Table 7 ). According to the multivariate regression model, stress when thinking during the course (C: 2.034; *p* = 0.006) and the baseline DASS anxiety score (C: 0.629; *p* < 0.001) were the most important factors affecting changes in DASS anxiety scores at follow-up ( Table 8 ). Increasing stress was associated with older age and stress when thinking about the course at baseline. Higher DASS anxiety scores at baseline reduced the increase in stress levels at follow-up and vice versa.

**Table 7:** Multivariate regression analysis with changes of DASS-S score as the dependent variable and target variables at baseline

Variable	C	95% CI LB	95% CI UB	*p* -Value
**Change in DASS-S**				
Gender (male)	−0.941	−2.297	0.415	0.172
Stress when thinking about the course (yes)	2.555	0.888	4.223	**0.003**
Stress due to COVID-19	0.036	0.012	0.060	**0.004**
DASS-S	0.515	0.357	0.673	**< 0.001**

Abbreviations: C, regression coefficient; CI, confidence interval; COVID-19, coronavirus disease 2019; DASS-S, Depression, Anxiety and Stress Scales stress; LB, lower boundary; UB, upper boundary.

Note: Significant *p* -values are marked in bold.

**Table 8:** Multivariate regression analysis with changes of DASS-A score as the dependent variable and target variables at baseline

Variable	C	95% CI LB	95% CI UB	*p* -Value
Change in DASS-A				
Age	−0.064	−0.259	0.132	0.520
Stress when thinking about the course (yes)	2.034	0.609	3.468	**0.006**
Course (clinical course)	−0.342	−1.544	0.859	0.573
DASS-A	0.629	0.471	0.788	**< 0.001**

Abbreviations: C, regression coefficient; CI, confidence interval; DASS-A, Depression, Anxiety and Stress Scales anxiety; LB, lower boundary; UB, upper boundary.

Note: Significant *p* -values are marked in bold.

## Discussion

The results of this study indicate that intraindividual differences in stress perception are more relevant to changes in stress and anxiety than course affiliation or the SARS-CoV-2 pandemic are in dental students. Thus, the study hypothesis was only partially confirmed.

In agreement with other studies that used different questionnaires, many participants showed medium stress levels during their holidays and during their course. 4 5 6 7 8 9 10 The mean DES scores did not change much during the study period and were comparable to those reported by Garbee et al. 21 Changes in DES scores between baseline and follow-up were affected by certain factors. For example, information about COVID-19 and DES scores at baseline affected the DES scores measured at follow-up. For the DASS questionnaire, stress when thinking during the course, stress due to COVID-19, and baseline stress scores were the most relevant factors for higher levels of general stress. These findings indicate that both individual stress perceptions and the SARS-CoV-2 pandemic might affect stress levels in dental students. This is in agreement with the results of Saraswathi et al and Hakami et al who also showed that the SARS-CoV-2 pandemic affected stress and anxiety levels in medicine and dental students. 24 25 However, the DASS questionnaire evaluates stress and anxiety in general, whereas the DES questionnaire evaluates stress specifically in dental students. It is therefore possible that the SARS-CoV-2 pandemic triggered general stress and not study-specific stress in our participants.

The salivary cortisol levels we observed in preclinical students were similar to those observed by Pani et al in final-year dentistry students in Saudi Arabia. 26 This increase in salivary cortisol observed in both studies can be explained by the pronounced workload of dentistry studies. 12 13 14 27 The SARS-CoV-2 pandemic may have increased stress levels in dental students during their clinical studies. Students have additional challenges during their clinical studies, such as dealing with patients. This exposure to patients increases their risk of being infected by droplet transmission 19 and being sent into quarantine because they are infected. This loss of study time could increase stress levels. However, cortisol levels did not increase during term time in students doing the clinical part of their course, and the mean levels even decreased slightly. This is surprising because cortisol levels were measured during winter in clinical students, when the spread of SARS-CoV-2 was higher, and during summer in preclinical students, when SARS-CoV-2 transmission was lower. Our finding that cortisol levels did not differ significantly between clinical and preclinical students during their course is in contrast to the findings of other studies that stress levels increased in clinical students. 8 16 In our study population, the individual perception of stress was more relevant to stress than course affiliation was.

### Strength and Weaknesses

Measuring salivary cortisol levels is a noninvasive and valid method for estimating stress levels. 28 29 30 31 However, cortisol levels vary during the day (high in the morning and low at night). 31 In this study, salivary cortisol was measured between 12:00 a.m. and 1:00 p.m. in all participants to get a reliable and constant view of the stress level. However, our results cannot be directly compared with those of all other studies because cortisol levels were measured at different times of day in different studies.

Another limitation of the study is the lack of data on stress levels in the participants before the SARS-CoV-2 pandemic. However, our results comparable to those of other studies that reported stress levels in dental students before the SARS-CoV-2 pandemic. Therefore, it may be possible to generalize our findings to the nonpandemic situation.

## Conclusion

Intraindividual differences in stress perception seem to be more relevant than course affiliation (preclinical or clinical) or the SARS-CoV-2 pandemic to changes in stress and anxiety levels among dental students. A follow-up study once the pandemic is over would be useful to determine stress levels in normal circumstances.
